# Paroxetine Increases δ Opioid Responsiveness in Sensory Neurons

**DOI:** 10.1523/ENEURO.0063-22.2022

**Published:** 2022-08-01

**Authors:** Allison Doyle Brackley, Nathaniel A. Jeske

**Affiliations:** 1 Departments of Physiology, University of Texas Health San Antonio, TX 78229; 2 Oral and Maxillofacial Surgery, University of Texas Health San Antonio, TX 78229; 3 Pharmacology, University of Texas Health San Antonio, TX 78229

**Keywords:** GRK2, opioid, pain, paroxetine

## Abstract

There are currently no Food and Drug Administration (FDA)-approved δ-opioid receptor (DOR)-selective agonists, despite having fewer side effects in rodents and nonhuman primates compared with traditional μ-opioid receptor (MOR) therapeutics (Vanderah, 2010). Targeting peripheral receptors is an attractive strategy to reduce abuse potential. However, peripheral opioid receptors do not readily respond to agonists unless primed by inflammation, which would limit their efficacy in noninflammatory pain patients (Stein et al., 1989). It was recently identified that G-protein-coupled receptor kinase 2 (GRK2) maintains DOR incompetence in noninflamed nociceptors (Brackley et al., 2016, 2017). Here, we report that paroxetine, a selective serotonin reuptake inhibitor (SSRI) and potent GRK2 inhibitor (Thal et al., 2012), reduces chronic GRK2 association with membrane DOR, thereby enhancing peripheral DOR-mediated analgesic competence in the absence of inflammation. Interestingly, paroxetine’s effects on GRK2 *in vivo* are limited to peripheral tissues in the male rat. The effects of paroxetine on DOR competence are notably antagonized by GRK2 overexpression. This is the first study to suggest that paroxetine induces peripheral DOR analgesic competence through a GRK2-dependent mechanism, improving analgesic efficacy in noninflamed tissue. Because paroxetine targets the protein that governs peripheral opioid receptor responsiveness, and does so in the absence of inflammation, we propose that paroxetine may be suitable as a co-therapy with peripherally-restrictive doses of opioids to improve analgesic efficacy in noninflammatory pain conditions.

## Significance Statement

Opioids that target MOR represent the gold-standard for analgesic health care, despite widespread abuse potential and the ongoing opioid-epidemic. Work herein uncovers the therapeutic potential of targeting peripheral δ-opioid receptor (DOR) for analgesic utility with a Food and Drug Administration (FDA)-approved G-protein-coupled receptor kinase 2 (GRK2) inhibitor paroxetine to boost efficacy and reduce side effect profiles. Analgesic pain management targeting DOR with increased efficacy through adjuvant paroxetine treatment could reduce over-reliance on MOR agonist opioids for pain relief and usher in new options for analgesia.

## Introduction

Opioids remain a pillar for severe pain management, despite debilitating central side effects that contribute to an ever-growing opioid epidemic in America. One analgesic strategy that has been gaining traction in recent years involves targeting peripheral opioid receptors to circumvent debilitating central side effects associated with systemically administered opioids. Traditional therapeutics systemically target μ-opioid receptor (MOR) and/or κ-opioid receptor (KOR) throughout the body. Interestingly, agonists that target the δ-opioid receptor Delta Opioid Receptor (DOR) produce analgesia as effectively as MOR agonists, but have a reduced side effect profile that includes less gastrointestinal dysfunction, tolerance, dependence and abuse potential (Vanderah, 2010). DOR is the most highly expressed opioid receptor subtype in peripheral pain-sensing neurons (Wang and Wessendorf, 2001; Bao et al., 2003). Perhaps for this reason, DOR has greater potential to treat inflammatory, thermal, mechanical and neuropathic pain modalities across genders compared with MOR and KOR, as evidenced by genetic deletion studies of individual opioid receptors (Nadal et al., 2006; Gavériaux-Ruff et al., 2008). However, peripheral DOR must be primed by inflammation before rendered analgesically competent (Stein et al., 1989; Patwardhan et al., 2005; Rowan et al., 2009; Brackley et al., 2016, 2017), which would limit the therapeutic efficacy of peripherally-restricted DOR agonist treatments alone to severe inflammatory pain patients only.

Mechanisms that regulate peripheral DOR incompetence have only recently been identified. *In vitro* and *in vivo* studies demonstrate that DOR incompetence in peripheral sensory neurons is governed by a chronic interaction between membrane-associated DOR and G-protein-coupled receptor kinase 2 (GRK2) maintained by constitutive protein kinase A (PKA)-dependent phosphorylation of GRK2 at Ser685 (Brackley et al., 2016, 2017). Novel therapies that disrupt these protein-protein interactions would be expected to enhance DOR functional competence in the periphery. The National Institutes of Health (NIH)’s National Center for Advancing Translational Sciences is currently pushing forward a new initiative that focuses on repurposing Food and Drug Administration (FDA)-approved drugs, supporting preclinical recycling of preexisting drugs for new indications that establish rationale for clinical trial implementation. In the past several years, the FDA-approved selective serotonin reuptake inhibitor (SSRI) paroxetine (Paxil) has been found to directly bind to GRK2, acting as a potent *in vitro* and *in vivo* inhibitor (Thal et al., 2012; Schumacher et al., 2015). In mice, paroxetine dose dependently enhances endogenous opioid antinociception (Gray et al., 1998; Kesim et al., 2005), mediated by DOR (Gray et al., 1998). In rats, paroxetine enhances antinociception produced by systemically-administered low-dose morphine, presumably targeting peripheral opioid receptors, in multiple pain modalities including neuropathic, mechanical, thermal, and cold allodynia (Lee et al., 2012). However, the mechanism that underlies paroxetine priming of opioid receptor analgesic competence has yet to be explored.

Given that paroxetine enhances opioid analgesia (Lee et al., 2012) and directly inhibits GRK2 (Thal et al., 2012), which constitutively maintains peripheral DOR incompetence (Brackley et al., 2016, 2017), we hypothesized that paroxetine-induced DOR competence in the periphery is mediated by sequestration of GRK2. This proof-of-concept study in male rats uses a drug recycling approach to target protein-protein interactions that govern peripheral opioid receptor responsiveness in the absence of inflammation. Using biochemical, molecular, functional, and behavioral techniques, we establish a physiological mechanism that provides rationale for a combination therapy between the SSRI paroxetine and peripherally-acting opioids. Repurposing this FDA-approved drug as an analgesic adjuvant would be expected to enhance opioid-mediated analgesia in noninflammatory pain patients with reduced incidence of debilitating systemic side effects.

## Materials and Methods

### Animals

Procedures using animals were approved by University of Texas Health Science Center at San Antonio Institutional Animal Care and Use Committee. Studies were conducted in accordance with the policies for the ethical treatment of animals established by the NIH with every effort made to limit animal discomfort and number of animals used.

### Neuronal cultures

For biochemistry, trigeminal ganglia (TG) were dissected bilaterally from adult male Sprague Dawley rats (200–250 g; Charles River Laboratories). TG were dissociated by collagenase treatment (30 min; Worthington), followed by trypsin treatment (30 min; Sigma-Aldrich). Dissociated TG were re-suspended in complete media (DMEM, Invitrogen Corp.) supplemented with 10% fetal bovine serum (FBS; Invitrogen), 100 ng/ml nerve growth factor (NGF; Harlan Laboratories), mitotic inhibitors (Sigma), 1% penicillin/streptomycin (Invitrogen), and 1% glutamine (Sigma) and plated on poly-D-lysine-coated plates (Corning). Similarly, for functional studies (Ca^2+^ imaging), dorsal root ganglia (DRG) dissected bilaterally at L4–L6 were dissociated by 40-min co-treatment with collagenase and dispase II (Sigma). Next, cells were re-suspended in complete media and plated on poly-D-lysine/laminin-coated coverslips (BD Biosciences). Cultures were maintained at 37°C and 5% CO_2_ and grown for 1–2 d for functional studies and 5–6 d for biochemistry with media changed the following day and every 2 d thereafter. TG were used for biochemical experiments to satisfy NIH requirements to reduce animal use in research.

### Cell/tissue processing and co-immunoprecipitation (Co-IP/IP)

Primary TG cultures were pretreated as indicated. Cells were harvested and homogenized in homogenization buffer [25 mm HEPES, 25 mm sucrose, 1.5 mm MgCl_2_, 50 mm NaCl (pH 7.4), 1 mm sodium pyrophosphate, 1 mm sodium orthovanadate (Sigma), 1 μg/ml pepstatin (Sigma), 1 μg/ml leupeptin (Sigma), 1 μg/ml aprotinin (Sigma), and 100 mm phenylmethylsulfonyl fluoride (PMSF; Sigma)] with 20 strokes using a Potter-Elvehjem pestle and glass homogenizer tube. Homogenates were placed on ice for 15 min and centrifuged at 1000 × *g* for 1 min to remove nuclei and un-lysed cells from the homogenate. Resulting supernatant was centrifuged at 16,000 × *g* for 30 min at 4°C to separate membrane proteins from cytosolic proteins. Cytosolic supernatant was separated from the pellet (crude membrane fraction), which was re-suspended in 250-μl homogenization buffer containing 1% Triton X-100 (Fisher Scientific).

For tissue analysis following *in vivo* injection, four tissues were collected following behavioral time course described below. TG and DRG (L4–L6) were bilaterally dissected, along with the corresponding lumbar spinal cord (SC). We also collected midbrain tissue that included midbrain and striato-pallidal regions. Dissected tissue samples were cut a minimum of 20 times, then homogenized in homogenization buffer with 40 strokes. As described above, homogenates were fractionated to separate membrane proteins from cytosolic proteins.

Total protein from membrane (PM) and/or cytosolic (CYTO) lysates were quantified (Bradford, Sigma), followed by Co-IP/IP^9-10^. Briefly, equal amounts of protein (125 μg) were immunoprecipitated with 1 μg anti-GRK2 (C-15, Santa Cruz Biotechnology) or anti-DOR (ab66317/ab66318, Abcam) antiserum for IP and Co-IP, respectively. Protein samples were eluted at 95°C for 5 min and placed at −20°C for Western blot (WB) analysis.

### WB analysis

Protein samples were resolved by 15% SDS-PAGE and transferred to polyvinyldifluoride membranes (PVDF; Millipore). Membranes were then blocked with 5% nonfat dried milk in Tris-buffered saline/Tween 20 (TBS-T: 15.35 mm Tris/HCl, 136.9 mm NaCl, pH 7.6, with 0.1% Tween 20) or 5% bovine serum albumin (Sigma) in TBS-T containing phosphatase inhibitor sodium orthovanadate (1 μm) for phosphorylation-specific antibodies. WBs were visualized using anti-phospho-GRK2-Ser685 (#12397-1, SAB Signalway Antibody; Brackley et al., 2017), anti-GRK2 (C-15, Santa Cruz Biotechnology; Wang et al., 2011), anti-DOR (ab66317/ab66318, Abcam; Brackley et al., 2016, 2017), anti-Caveolin-1 (N-20, Santa Cruz Biotechnology; Gomez et al., 2011), anti-β1-integrin (Santa Cruz Biotechnology; Por et al., 2012), or anti-β-actin (I-19-R, Santa Cruz Biotechnology; Gomez et al., 2011), followed by appropriate horseradish-peroxidase-conjugated secondary antisera (GE Healthcare) and ECL (enhanced chemiluminescence or prime) detection following the manufacturer’s protocol (GE Healthcare). Antibody specificities were verified by the manufacturers, BLAST sequence analysis, and used in previous publications as indicated. Integrated density measurement values, equivalent to the product of area and mean gray value by histogram analysis, were performed using NIH ImageJ software.

### siRNA transfection

Specific FITC-labeled siRNA duplexes custom-designed to target GRK2 were previously designed and characterized (QIAGEN; Brackley et al., 2016). The sequence for the sense strand of GRK2 siRNA was 5′-GCAGAAGUAUCUAGAGGAUUU-3′ and antisense strand of GRK2 siRNA was 5′-AUCCUCUAGAUACUUCUGCUU-3′. For Ca^2+^ imaging experiments, FITC-labeled siRNA duplexes (45 ng/coverslip) were transfected into cultured sensory neurons using HiPerFect (QIAGEN), following manufacturer’s directions as described previously (Brackley et al., 2016). Additional cells were treated with no siRNA (mock), used as a negative control.

### cDNA nucleofection

DRG were cultured as described above and nucleofected with Effectene nucleofection reagent (QIAGEN) following manufacturer’s instruction, maintaining a cDNA to enhancer reagent ratio of 1:8, for 10 h, as described previously (Brackley et al., 2016). For these experiments, empty vector (E.V.) pcDNA3.1 or GRK2 (Jeffrey L. Benovic, Thomas Jefferson University) cDNA (500 ng/coverslip) were nucleofected into sensory neurons. GFP cDNA (250 ng/coverslip) was co-nucleofected to identify positive transfection.

### Single-cell Ca^2+^ imaging

Following 2-h serum-starvation, cultured DRG were loaded with fura-2 A.M. (1 μm; Invitrogen) and pluronic F-127 (0.04%; Invitrogen) for 1 h at 37°C in the dark, in standard extracellular solution (SES) containing: 140 mm NaCl, 4 mm KCl, 2 mm CaCl_2_, 1 mm MgCl_2_, 10 mm HEPES, and 10 mm D-(+)-glucose, pH 7.40. Neurons were observed on an inverted Nikon Eclipse T_i_-U microscope fitted with a 20×/0.75 numerical aperture Fluor objective and imaged using MetaFluor System for Ratio Fluorescence (MetaMorph). Fluorescent images were taken as previously described (Brackley et al., 2016). The following criteria were used to indicate positive sensory neuronal phenotype within a heterogenous culture: (1) bright round cell bodies with clear nuclei (Goldenberg and De Boni, 1983; Liu et al., 2013); (2) depolarization in response to 50 mm KCl (Khasabova et al., 2004; Pettinger et al., 2013); and (3) sensitivity to capsaicin [CAP; 1 μm; 25% above baseline (BL), Sigma; Brackley et al., 2016]. Corresponding filters were used to restrict analysis to FITC-siRNA-positive or GFP-positive DRG.

DOR activity was quantified as a measure of [D-Pen^2,5^]-enkephalin (DPDPE, 1 μm; Sigma) inhibition of 50 mm KCl-evoked Ca^2+^ transients in CAP (1 μm)-sensitive DRG as previously described (Brackley et al., 2016). A perfusion valve controller (<0.1 psi) and multi-barrel glass pipette were used to apply 3 s exposures of KCl at 60 s, 270 s, 740 s, 950 s, and 1440 s, followed by CAP at 1770 s. To assess DOR competence, the first two exposures to KCl were in SES and the second two in the presence of bath-perfused DPDPE, followed by a fifth exposure to KCl following DPDPE washout to confirm neuron remained viable. In CAP-sensitive DRG, single-cell recording traces were used to obtain the area under the curve (AUC), calculated as an integral across 3 min for the average response to KCl in the absence or presence of a DOR agonist. The following equations were used to calculate DOR activity:

$$KCl=\frac{\left(KCl_{1},KCl_{2}\right)}{3}$$

$$KCl+DPDPE=\frac{\left(KCl_{3},KCl_{4}\right)}{2}$$

$$DPDP\;Agonist\;Inhibition\left(\%\right)=100-(\frac{AUC_{KCl+DPDPE}}{AUC_{KCl}}\times 100\%).$$

For Ca^2+^ imaging, stock solution of DOR agonist DPDPE (1 mm) was prepared in sterile water. CAP (1 mm) stock solution was prepared in EtOH. Paroxetine (25 mm; Sigma/Toronto Research Chemicals), CMPD101 (2.5 mm; Tocris), and fluoxetine hydrochloride (25 mm; Sigma) stock solution was prepared in DMSO. KCl was prepared fresh in SES the day of experimentation. All drugs and appropriate vehicles were diluted in SES to doses determined by IC_50_ values in this study and others. The IC_50_ value of paroxetine concentration response curve (3.8 μm) yielded similar values to that observed for GRK2 inhibition (4.7 μm; Thal et al., 2012), 5 μm was the *in vitro* dose used for paroxetine the remainder of the study. GRK2/3 inhibitor CMPD101, which binds the same site as paroxetine, served as a positive control (Thal et al., 2011). Because doses >2 μm CMPD101 reduces selectivity for GRK2 over PKA, 0.5 μm was for *in vitro* studies. Fluoxetine, a typical SSRI that does not inhibit GRK2 at doses exceeding 100 μm, was used as a negative control at equimolar doses to paroxetine (Thal et al., 2012).

### Behavioral test for paroxetine priming of DOR functional competence

Electronic von Frey (IITC Life Science Inc.) was used to assess mechanical allodynia in male Sprague Dawley rats (250–300 *g*), measured by paw withdrawal threshold (PWT; g) to a rigid tip. Rats were given 2 h to acclimate to environment and 30 min to the raised von Frey mesh stand. For testing, the rigid tip was applied to the hind paw. Upon reaction, the system displayed an electronic reading for PWT. A minimum of six BL readings were taken from ipsilateral and contralateral hind paws with duplicate measurements for each time point, then averaged for statistical analysis.

DOR analgesic competence was measured as DPDPE (20 μg) inhibition of prostaglandin E_2_ (PGE_2_; 0.3 μg; Cayman Chemicals)-induced allodynia (Rowan et al., 2009; Brackley et al., 2016). Following BL readings, animals were injected intraperitoneally with vehicle [10% dimethylsulfoxide (DMSO)/90% Dulbecco’s PBS (DPBS)], paroxetine (5 mg/kg), CMPD101 (0.5 mg/kg), or fluoxetine (5 mg/kg); 25 min after intraperitoneal injection, an intraplantar (i.pl.) co-injection of DPDPE and PGE_2_ was given into the hind paw. PWT readings were recorded by a blinded observer every 5 min for 20 min following each injection. Intraperitoneal drugs were administered at a final volume of 1 ml and i.pl. co-injection at a final volume of 50 µl.

### Statistics

GraphPad Prism 5.0 was used for statistical analyses (GraphPad Software,). Quantitative data expressed as mean ± SEM. Statistical significance was determined by one-way ANOVA or two-way ANOVA with Bonferroni *post hoc* analyses as needed; *p* < 0.05 was considered statistically significant.

## Results

### Paroxetine reduces GRK2 association with DOR to enhance receptor competence

**Figure 1.**
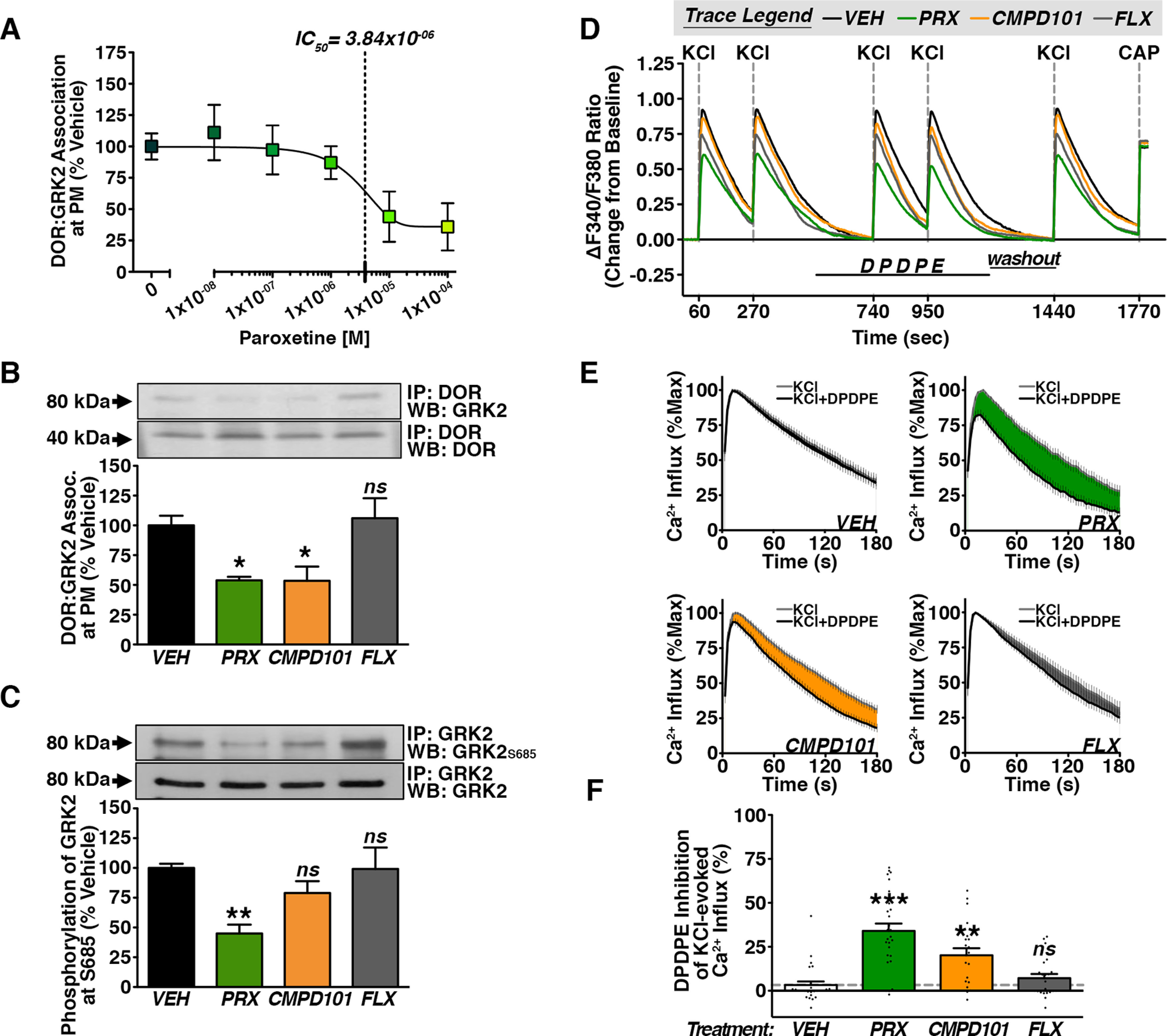
Paroxetine enhances Delta Opioid Receptor (DOR) functional competence in sensory neurons. ***A***, Concentration response curve for paroxetine ([M], 10 min) effect on GRK2 Co-IP with DOR in crude membrane fractions from serum-starved TG cultures [IC_50_ = 3.840 μm (vertical black dotted line), least squares fit (best-fit) variable slope curve (black line), ANOVA summary: *F*_(5,18)_ = 2.932, *p* = 0.415, one-way ANOVA with Bonferroni *post hoc*, mean ± SEM, *n* = 4 independent trials from 24 TG/12 total rats]. ***B***, GRK2 Co-IP with membrane-associated DOR in serum-starved TG cultures treated for 10 min with vehicle (VEH; DMSO), paroxetine (PRX; 5 μm), CMPD101 (0.5 μm), or fluoxetine (FLX; 5 μm; *p < 0.05, ns = not significant, ANOVA summary: *F*_(3,12)_ = 8.805, *p* = 0.0023, one-way ANOVA with Bonferroni *post hoc*, mean ± SEM, *n* = 4 independent trials from 24TG/12 total rats). ***C***, GRK2 phosphorylation at Ser685 isolated by GRK2 IP in serum-starved TG cultures treated for 10 min with vehicle (VEH; DMSO), paroxetine (PRX; 5 μm), CMPD101 (0.5 μm), or fluoxetine (FLX; 5 μm; **p < 0.01, ns = not significant, ANOVA summary: *F*_(3,12)_ = 7.328, *p* = 0.0047, one-way ANOVA with Bonferroni *post hoc* test, mean ± SEM, *n* = 4 independent trials from 24 TG/12 total rats). ***D–F***, Cumulative (***C***) mean experimental traces, (***D***) average KCl response traces, and (***E***) quantification of DPDPE (1 μm) inhibition of KCl (50 mm)-evoked Ca^2+^ influx in CAP (1 μm)-sensitive serum-starved DRG pretreated for 10 min with vehicle (VEH; DMSO), paroxetine (PRX; 5 μm), CMPD101 (0.5 μm), or fluoxetine (FLX; 5 μm; ***p < 0.005, **p < 0.01, ns = not significant, ANOVA summary: *F*_(3,92)_ = 18.83, *p* < 0.0001, one-way ANOVA with Bonferroni *post hoc*, mean ± SEM, *n* = 21–27 DRG/group collected from a minimum of 5 rats).

Previous studies establish that the SSRI paroxetine directly interacts with and inhibits GRK2, which promotes G-protein-coupled receptor (GPCR) signaling *in vitro* and *in vivo* (Thal et al., 2012; Schumacher et al., 2015). Under naive conditions, GRK2 constitutively associates with membrane-bound DOR, rendering the receptor unresponsive to agonist stimulation in peripheral sensory neurons (Brackley et al., 2016, 2017). Given that paroxetine-induced analgesia can be dose dependently antagonized by a DOR-selective antagonist (Gray et al., 1998; Kesim et al., 2005), we sought to determine whether paroxetine disrupts membrane DOR-GRK2 association. Paroxetine dose dependently reduces GRK2 Co-IP with DOR in peripheral sensory neuron membrane fractions (Fig. 1*A*). Additional controls monitored whether paroxetine’s effects on GRK2 are shared by a chemically related compound, CMPD101, a selective GRK2 and GRK3 inhibitor, or unrelated compound, fluoxetine, in the same drug class (Fig. 1*B*). Compared with vehicle-treated cells, GRK2 association with membrane-bound DOR is reduced to 55.48 ± 3.46% and 53.62 ± 17.89% by paroxetine and CMPD101, respectively. In contrast, fluoxetine yields levels comparable to vehicle. These data verify that paroxetine reduces GRK2 association with DOR in peripheral sensory membrane fractions.

In peripheral sensory neurons, PKA-dependent phosphorylation of GRK2 drives the constitutive association between membrane-bound DOR and GRK2 (Brackley et al., 2017). Therefore, we tested whether paroxetine affects PKA-dependent phosphorylation of GRK2 at Ser685 in immunoprecipitated lysates using a site-specific antibody. Paroxetine treatment reduces GRK2 phosphorylation at Ser685 to 45.50 ± 11.09% of vehicle-treated cells. Whereas, CMPD101 reduces PKA-dependent GRK2 phosphorylation by about half that to 74.40 ± 12.58%. Again, fluoxetine yields results comparable to vehicle (Fig. 1*C*). Thus, paroxetine reduces PKA phosphorylation of GRK2 at Ser685 in peripheral nociceptors.

To build on this, functional experiments were conducted to determine whether paroxetine enhances functional DOR competence. For this purpose, we measured opioid inhibition of voltage-gated Ca^2+^ channels (VGCCs) evoked by KCl-induced Ca^2+^ influx in cultured sensory neurons (Brackley et al., 2016, 2017). Given that paroxetine reduces phosphorylation of GRK2 at Ser685, an important site for GRK2 maintenance of functional DOR incompetence (Brackley et al., 2017), we next tested whether paroxetine enhances DOR responsiveness to agonist stimulation. Recapitulating published population findings in primary sensory neurons, DOR agonist DPDPE, which has ∼100-fold selectivity for DOR over other opioid receptor subtypes, fails to inhibit KCl-evoked Ca^2+^ transients in CAP-sensitive DRG (Fig. 1*D–F*). Paroxetine pretreatment, on the other hand, produces a robust response to DPDPE and inhibits KCl-evoked Ca^2+^ transients collectively by 34.59 ± 4.30%. Pretreatment with CMPD101 produces DPDPE inhibition of KCl-evoked Ca^2+^ transients by 20.19 ± 4.11%. Fluoxetine, which does not reduce PKA-dependent phosphorylation of GRK2, simultaneously fails to enhance DOR competence in peripheral sensory neurons (Fig. 1*D–F*). Taken together, paroxetine enhances DOR functional competence in CAP-sensitive sensory neurons.

### Paroxetine enhances functional receptor competence by antagonizing GRK2-DOR interaction

**Figure 2.**
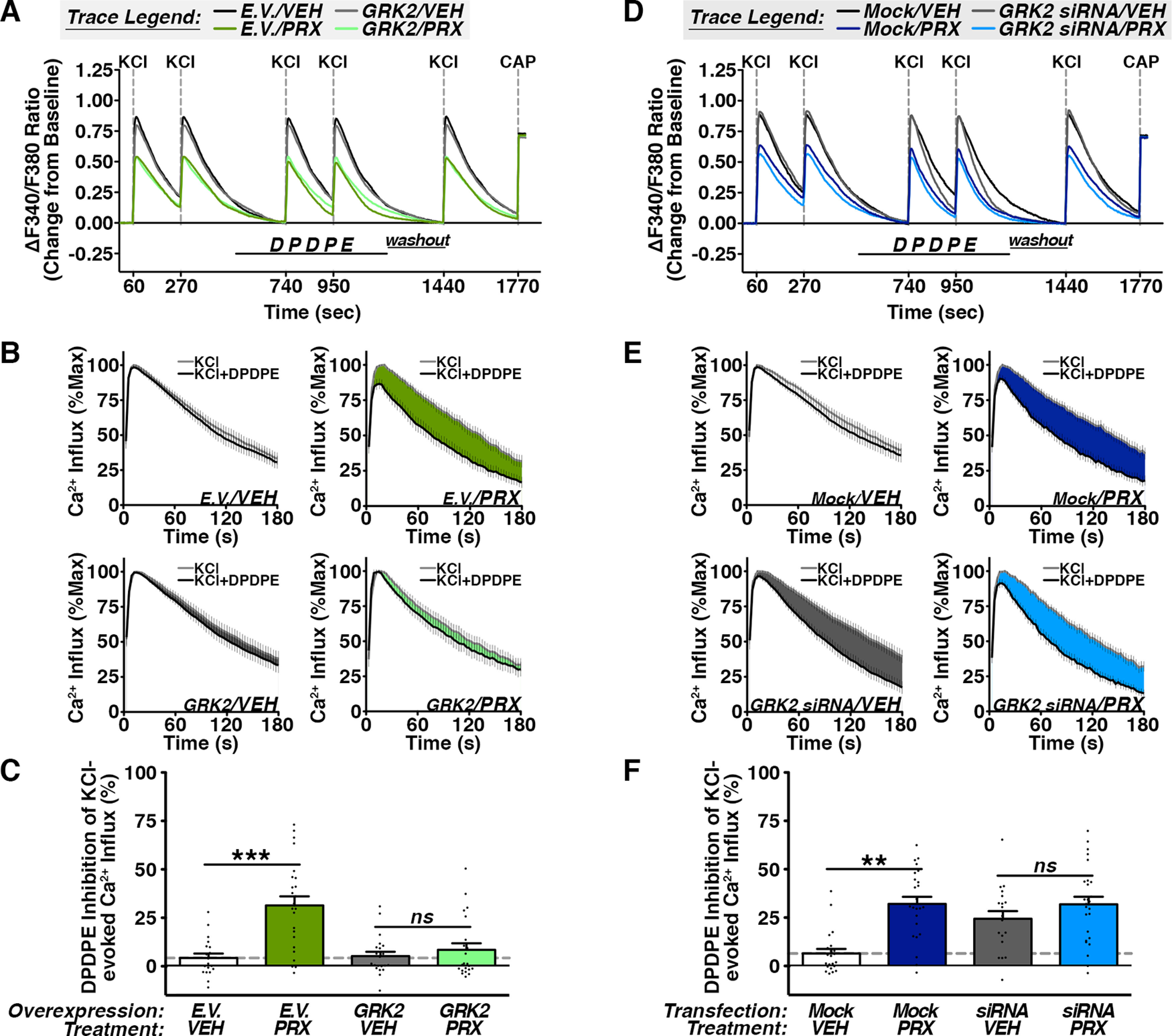
Paroxetine enhances Delta Opioid Receptor (DOR) functional competence via GRK2-dependent mechanism. ***A–C***, Cumulative (***A***) mean experimental traces, (***B***) average KCl response traces, and (***C***) quantification of DPDPE (1 μm) inhibition of KCl (50 mm)-evoked Ca^2+^ influx in CAP (1 μm)-sensitive serum-starved nucleofected DRG pretreated with vehicle (VEH; DMSO) or paroxetine (PRX; 5 μm) for 10 min [***p ≤ 0.005, ns = not significant, ANOVA summary: Interaction: *F*_(1,82)_ = 11.72, *p* = 0.0010, Overexpression (E.V. vs GRK2): *F*_(1,82)_ = 9.900, *p* = 0.0023, Pretreatment (VEH vs PRX): *F*_(1,82)_ = 19.12, *p* < 0.0001, two-way ANOVA with Bonferroni *post hoc*, mean ± SEM, *n* = 19–24 DRG/group collected from a minimum of 5 rats]. ***D–F***, Cumulative (***D***) mean experimental traces, (***E***) average KCl response traces, and (***F***) quantification of DPDPE (1 μm) inhibition of KCl (50 mm)-evoked Ca^2+^ influx in CAP (1 μm)-sensitive serum-starved transfected DRG pretreated with vehicle (VEH; DMSO) or paroxetine (PRX; 5 μm) for 10 min [**p ≤ 0.01, ns = not significant, ANOVA summary: Interaction: *F*_(1,87)_ = 6.377, *p* = 0.0134, Transfection (Mock vs FITC-GRK2 siRNA): *F*_(1,87)_ = 6.141, *p* = 0.0151, Pretreatment (VEH vs PRX): *F*_(1,87)_ = 21.27, *p* < 0.0001, two-way ANOVA with Bonferroni *post hoc*, mean ± SEM, *n* = 19–26 DRG/group collected from a minimum of 5 rats].

In sensory neurons, paroxetine reduces DOR association with GRK2 by way of reduced PKA-dependent phosphorylation of GRK2 and, thus, increases functional DOR competence (Fig. 1). Because paroxetine binds GRK2 (Thal et al., 2012), we hypothesized that paroxetine mechanistically targets GRK2 to functionally enhance DOR competence. To assess this, we overexpressed GFP with E.V. or wild-type GRK2 and measured DOR responsiveness to DPDPE in pretreated with vehicle or paroxetine. GFP-positive CAP-sensitive DRG nucleofected with E.V. or GRK2 are similarly insensitive to DPDPE, producing only 4.26 ± 2.27% and 5.22 ± 2.22% inhibition of KCl-evoked Ca^2+^ transients, respectively (Fig. 2*A–C*). Paroxetine pretreatment in control DRG produces significant DPDPE sensitivity and collectively inhibits KCl-induced Ca^2+^ influx by 31.37 ± 4.69%. However, overexpression of GRK2 in CAP-sensitive DRG paroxetine fails to induce functional DOR competence with only 8.52 ± 3.35% DPDPE inhibition of KCl-evoked Ca^2+^ transients (Fig. 2*A–C*). Thus, GRK2 overexpression antagonizes paroxetine-induced DOR competence in primary sensory neurons.

Paroxetine, like most clinically efficacious SSRI’s, has off-target affinity for receptors and proteins other than the serotonin transporter (Owens et al., 1997, 2002; Thal et al., 2012). To ascertain whether a GRK2-independent mechanism may account for any paroxetine-induced DOR competence in sensory neurons, we conducted the same Ca^2+^ imaging paradigm following GRK2 knock-down in DRG using previously validated FITC-GRK2 siRNA (Brackley et al., 2016). In line with published population findings in primary cultures, vehicle pretreatment in mock-treated DRG yields only 6.44 ± 2.35% DPDPE inhibition of KCl-evoked Ca^2+^ influx (Fig. 2*D–F*). On the other hand, mock-treated DRG exposed to paroxetine collectively produce a significant response to DPDPE and inhibit KCl-evoked Ca^2+^ influx by 32.05 ± 3.67%. DRG transfected with FITC-GRK2 siRNA robustly respond to the DOR agonist DPDPE and readily inhibits KCl-evoked Ca^2+^ transients by 24.40 ± 3.96% following vehicle pretreatment. Similarly, DPDPE inhibits 31.88 ± 3.88% of KCl-evoked Ca^2+^ transients in FITC-GRK2 siRNA DRG following paroxetine pretreatment (Fig. 2*D–F*). Notably, in GRK2 knock-down DRG, paroxetine pretreatment does not significantly improve DOR competence in vehicle-treated neurons. Taken together with our biochemical observations, data presented herein identify that paroxetine-induced DOR competence is driven by its sequestration of GRK2 from membrane-bound DOR in sensory neurons.

### Paroxetine enhances DOR-mediated analgesia *in vivo* by targeting peripheral GRK2

**Figure 3.**
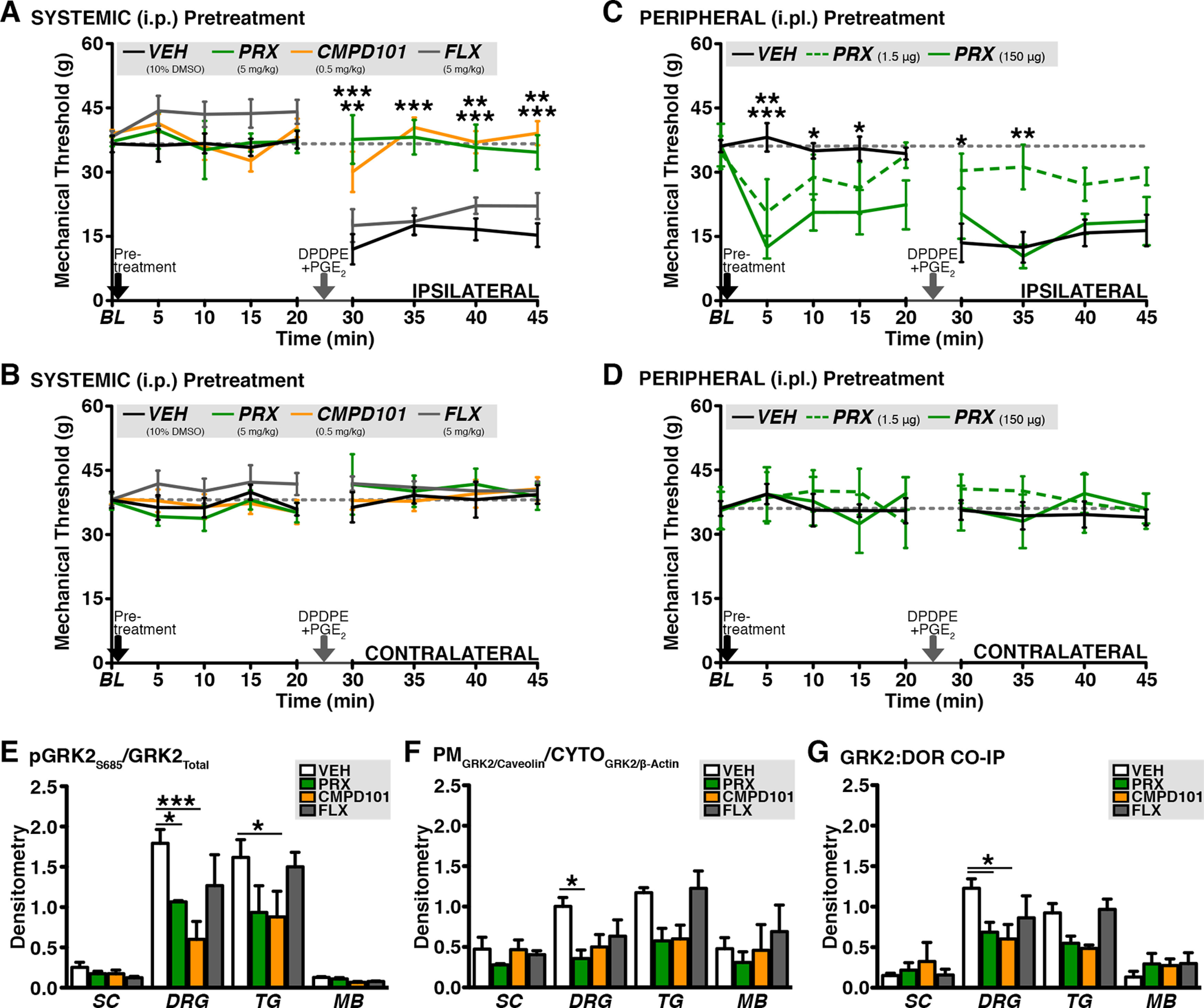
Paroxetine targets GRK2 to modulate of DOR-mediated antinociception. ***A***, ***B***, DPDPE (20 μg) inhibition of PGE_2_ (0.3 μg)-induced mechanical allodynia in (***A***) ipsilateral and (***B***) contralateral hindpaws following systemic treatment. Readings were collected at 5-min intervals for 20 min following initial (intraperitoneal) injection [black arrow, vehicle (VEH; 10%DMSO/90%DPBS), paroxetine (PRX; 5.0 mg/kg), CMPD101 (0.5 mg/kg), or fluoxetine (FLX; 5.0 mg/kg)] and a second (i.pl.) injection (gray arrow, co-injection DPDPE (20 μg)/PGE_2_ (0.3 μg); DPDPE inhibition of PGE_2_-induced allodynia: ***A***, VEH versus PRX (***p < 0.005 at 30, 35 min, **p < 0.01 at 40, 45 min) or CMPD1010 (**p < 0.01 at 30 min, ***p < 0.005 at 35, 40, 45 min), FLX versus PRX (**p < 0.01 at 30 and ***p < 0.005 at 35) and CMPD101 (***p < 0.005 at 35); ipsilateral ANOVA summary: Interaction: *F*_(24,180)_ = 4.358, *p* = 0.0001, Treatment: *F*_(3,180)_ =19.16, *p* = 0.0001, Time: *F*_(8,180)_ = 14.72, *p* = 0.0001; repeated measures two-way ANOVA Bonferroni *post hoc*; mean ± SEM, *n* = 6 rats/group. ***C***, ***D***, DPDPE (20 μg) inhibition of PGE_2_ (0.3 μg)-induced mechanical allodynia in (***C***) ipsilateral and (***D***) contralateral hindpaws following the timeline described above, but initial injection was peripherally-administered [i.pl., VEH or PRX (1.50 μg or 150 μg); ***C***, PRX-induced allodynia: VEH vs 1.50 μg (**p < 0.01 at 5 min) or 150 μg (***p < 0.005 at 5 min; *p < 0.05 at 10–15 min); DPDPE inhibition of PGE_2_-induced allodynia: VEH vs 1.50 μg (*p < 0.05 at 30 min, **p < 0.01 at 35 min); ipsilateral ANOVA summary: Interaction: *F*_(16,135)_ = 2.915, *p* = 0.0004, Treatment: *F*_(2,135)_ = 11.21, *p* = 0.0001, Time: *F*_(8,135)_ = 5.410, *p* = 0.0001; repeated measures two-way ANOVA Bonferroni *post hoc*; mean ± SEM, *n* = 6 rats/group]. ***E–G***, Molecular changes 50 min following systemic (intraperitoneal) injection [vehicle (VEH; 10%DMSO/90%DPBS), paroxetine (PRX; 5.0 mg/kg), CMPD101 (0.5 mg/kg), or fluoxetine (FLX; 5.0 mg/kg)] in spinal cord (SC), dorsal root ganglia (DRG), trigeminal ganglia (TG), and midbrain (MB)]. PKA-dependent phosphorylation of GRK2 at Ser685 (***E***), GRK2 translocation (***F***), and GRK2 association with membrane-bound DOR [***G***; *p < 0.05, **p < 0.01, ANOVA summary (phosphoGRK2-Ser685): Interaction: *F*_(9,27)_ = 1.830, *p* = 0.1085, Tissue: *F*_(3,9)_ = 25.17, *p* = 0.0001, Treatment: *F*_(3,27)_ = 8.393, *p* = 0.0004, Matching: *F*_(9,27)_ = 2.272, *p* = 0.0480, ANOVA summary (GRK2 translocation): Interaction: *F*_(9,27)_ = 1.441, *p* = 0.2202, Tissue: *F*_(3,9)_ = 4.558, *p* = 0.0332, Treatment: *F*_(3,27)_ = 7.930, *p* = 0.0006, Matching: *F*_(9,27)_ = 3.103, *p* = 0.0108, ANOVA summary (DOR:GRK2 association): Interaction: *F*_(9,27)_ = 2.025, *p* = 0.0758, Tissue: *F*_(3,9)_ = 5.469, *p* = 0.0204, Treatment: *F*_(3,27)_ = 3.468, *p* = 0.0299, Matching: *F*_(9,27)_ = 4.691, *p* = 0.0008, *n* = 3–4 independent trials of tissue collected from 16 total rats, matched two-way ANOVA with Bonferroni correction]. See Extended Data Figure 3-1 for representative WB images.

Paroxetine’s selective interaction with GRK2 *in vitro and in vivo* enhances GPCR signaling (Thal et al., 2012; Schumacher et al., 2015). After we identified that paroxetine facilitates opioid signaling in sensory neurons *in vitro*, we measured its physiological effect on peripheral DOR-mediated analgesia *in vivo*. To assess whether systemic (Fig. 3*A*,*B*) or peripheral (Fig. 3*C*,*D*) paroxetine pretreatment enhances DOR analgesic competence, we assessed DPDPE inhibition of PGE_2_-induced mechanical allodynia in rats. In vehicle (intraperitoneally, 10% DMSO/90% DPBS)-treated animals, a peripherally restrictive dose of DPDPE (20 μg, i.pl.; Rowan et al., 2009) co-injected into the hind paw does not elicit DOR-mediated analgesia, which is measured as neutralization of PGE_2_ (0.3 μg, i.pl.)-induced allodynia. However, paroxetine (5 mg/kg, i.p.) and CMPD101 (0.5 mg/kg, i.p.) systemically produce equivalent DOR-mediated analgesia *in vivo,* as measured by a return to BL in the ipsilateral hind paw. Congruent with biochemical and functional data, fluoxetine (0.5 mg/kg, i.p.) fails to reverse peripheral DOR incompetence (Fig. 3*A*). Interestingly, a similar dose of paroxetine (150 µg, i.pl.) injected into the hind paw produces allodynia and edema, whereas a dose two log units lower (1.50 µg, i.pl.) not only produces less allodynia, but primes DOR analgesic competence (Fig. 3*C*). No changes were observed across treatment groups in contralateral hind paws (Fig. 3*B*,*D*). These data demonstrate that systemic paroxetine treatment reverses peripheral DOR analgesic incompetence *in vivo*. The results of these experiments also suggest that concentrated local inhibition of GRK2 produces allodynia that supersedes DOR-mediated analgesia.

10.1523/ENEURO.0063-22.2022.f3-1 Extended Data Figure 3-1 ***A***, Representative WB images used to calculate values in Figure 3*E*. ***B***, Representative WB images used to calculate values in Figure 3*F*. ***C***, Representative WB images used to calculate values in Figure 3*G*. Download Figure 3-1, TIF file.

Next, we sought to determine whether drug-induced molecular changes occur at the doses that result in DOR-mediated analgesia, peripheral (TG, DRG) and central tissues (brain, SC). Based on posttranslational changes governing functional DOR competence (Brackley et al., 2017), we assessed PKA-mediated phosphorylation of GRK2 (Fig. 3*E*), membrane-targeting of GRK2 (Fig. 3*F*), and GRK2 association with plasma membrane DOR (Fig. 3*G*). Surprisingly, paroxetine only induced changes in peripheral tissues (DRG and TG), not central (SC or midbrain; Fig. 3*E*,*F*). In TG and DRG, only paroxetine and CMPD101 significantly reduce GRK2 phosphorylation at Ser685 and GRK2 translocation to the plasma membrane. Accordingly, similar effects occur in respect to GRK2 association with membrane-bound DOR with significant reductions on GRK2-DOR association in DRG (Fig. 3*G*). Collectively, these findings suggest that paroxetine reverses peripheral opioid receptor incompetence selectively through changes to GRK2 in the periphery.

## Discussion

Efforts to manage severe pain without disabling side effects including respiratory depression, tolerance and substance abuse, increasingly point toward peripheral opioid receptors as potential therapeutic targets. One challenge to this approach is that these receptors are analgesically incompetent unless primed by inflammation, which is conserved in animals (Stein et al., 1989; Obara et al., 2009; Rowan et al., 2009; Brackley et al., 2016) and humans (Stein et al., 1991; Likar et al., 2001). Despite the analgesic potential of this receptor subpopulation, the therapeutic effectiveness of peripherally-restrictive opioid agonists would be limited to severe inflammatory pain. The catalyst that restores functional opioid receptor competence depends on the induction of a signaling cascade that results in the summation of GRK2 sequestration from the receptor (Brackley et al., 2016). FDA-approved drug paroxetine, which binds and inhibits GRK2 (Thal et al., 2012), may have analgesic potential by enhancing peripheral opioid receptor competence in multiple pain modalities (Lee et al., 2012). In this study, we identify a GRK2-dependent mechanism that establishes a rationale for clinical trial implementation of repurposing of the FDA-approved drug paroxetine as a potential co-treatment to restore peripheral opioid competence.

Paroxetine has long been FDA approved and is currently indicated for the treatment of major depressive disorder, obsessive compulsive disorder, panic disorder, social anxiety disorder, generalized anxiety disorder, posttraumatic stress disorder, and was recently approved to treat vasomotor symptoms associated with menopause. Although not currently indicated for the treatment of pain, multiple studies indicate that paroxetine, on its own or as a co-therapy, is analgesically efficacious for a variety of human pain conditions. These pain conditions include headache and migraine (Foster and Bafaloukos, 1994; Langemark and Olesen, 1994; Holroyd et al., 2003; Park et al., 2006; Davanzo et al., 2014), fibromyalgia (Patkar et al., 2007; Pae et al., 2009a,b; Ramzy, 2017), diabetic neuropathy (Sindrup et al., 1990), irritable bowel syndrome (Marks et al., 2008), burning mouth syndrome (Maina et al., 2002), rheumatoid arthritis (Bird and Broggini, 2000), temporomandibular disorder (Inagaki et al., 2007), noncardiac chest pain (Doraiswamy et al., 2006), phantom limb pain (Nagoshi et al., 2012), and somatic pain comorbid with the disorders for which paroxetine is indicated (Aikens et al., 2008; Wise et al., 2008; Hollander et al., 2010). However, one lumbar chronic back pain study found a nonsignificant 25% reduction in pain intensity (Atkinson et al., 1999), and case studies reveal that paroxetine may not be appropriate for rare pain conditions (Zhu et al., 2008). Nevertheless, overwhelming evidence supports paroxetine’s potential to be repurposed for the treatment of pain.

Paroxetine human studies vary greatly in dose and treatment time. Pharmacokinetic properties of paroxetine were investigated in humans before its approval by the FDA^57-60^ (Lund et al., 1979, 1982; Greb et al., 1989; Kaye et al., 1989). Paroxetine is highly bioavailable and undergoes quick, extensive first-past metabolism. Its lipophilic properties facilitate wide distribution throughout the body with only 1% remaining in the plasma. Of the preclinical studies for its use in human pain conditions, plasma levels of paroxetine have only been reported for diabetic neuropathy (Sindrup et al., 1990). Interestingly, paroxetine treatment at doses 10–50 mg requires plasma levels of 200–660 and 300–800 nm, respectively, for maximal relief in most patients. This dose range is within one log unit of our concentration response curve (Fig. 1*A*). Given that paroxetine can ipsilaterally affect proteins, including GRK2, in the periphery under painful conditions (Gai et al., 2014), it is likely that a substantial portion of the remaining 99% of paroxetine is present at higher concentrations locally in tissues that require it. Indeed, human brain SSRI concentrations up to 20:1 compared with plasma levels have been reported (Karson et al., 1993). Thus, the paroxetine concentration that targets GRK2 and prime the peripheral opioid system in animals may be within the physiological range present in tissues of human pain patients.

This study was performed in male rats and, given that GRK2 phosphorylation is reportedly higher in female rodents (Abraham et al., 2018), paroxetine could be more therapeutically effective at increasing peripheral DOR competence in females compared with males. Indeed, selective GRK2 inhibition in female mice increased analgesic effects of systemic MOR and KOR agonists (Abraham et al., 2018). These findings warrant additional studies in female rodents to determine whether paroxetine could maximally prime the peripheral opioid system, circumventing estrogen regulation of GRK2.

Although highly selective for GRK2/3, CMPD101 is less selective of GRK2 over GRK3 (Thal et al., 2011) compared with paroxetine (Thal et al., 2012). Both GRK inhibitors were able to attenuate GRK2 association with membrane-bound DOR (Fig. 1*B*) and enhance DPDPE inhibition of KCl-evoked Ca^2+^ influx (Fig. 1*D–F*). Although there were no significant differences observed between paroxetine and CMPD101, it is apparent in the summary data that paroxetine showed both a greater inhibition of GRK2 phosphorylation at Ser365 (Fig. 1*C*) and DPDPE inhibition of KCl-evoked Ca^2+^ influx (Fig. 1*F*). Therefore, CMPD1010 cross-inhibition of GRK3, which is also expressed in sensory neurons (Diverse-Pierluissi et al., 1996), could explain the subtle differences observed between GRK inhibitors in these cultured neuron experiments.

CMPD101 largely produces analogous effects to paroxetine *in vivo* (Fig. 3) and in sensory neuron cultures (Fig. 1) and tissue (Fig. 3), but we identify only one functional similarity between the SSRIs fluoxetine and paroxetine. Each partially inhibits VGCCs in the absence of opioid ligand (Fig. 1*D*). While such a phenomenon has been reported in neurons of the central nervous system (Stauderman et al., 1992; Deák et al., 2000), to our knowledge, this is the first report of such an effect in peripheral sensory neurons. According to our data, this does not predict the priming capacity of functional (Fig. 1*D–F*) or physiological (Fig. 3*A*) DOR competence. Given that paroxetine’s effect on KCl-evoked Ca^2+^ transients in the absence of opioid was unchanged following GRK2 knock-down or overexpression (Fig. 2*A*,*D*), this SSRI effect is GRK2-independent. Interestingly, paroxetine’s effect is twice that of fluoxetine at equimolar doses. This may be due, in part, to its unique molecular structure relative to other SSRIs. Paroxetine is a phenylpiperidine and nearly all phenylpiperidines used clinically in research capacities are opioids. Interestingly, this structure is part of the morphine and fentanyl molecules. Furthermore, paroxetine-induced analgesia is dose dependently reversed by opioid receptor antagonists (Gray et al., 1998; Kesim et al., 2005). This raises the possibility that paroxetine may have some affinity for opioid receptors in addition to its known target proteins (Owens et al., 1997, 2002; Thal et al., 2012).

In conclusion, experimental results demonstrate that paroxetine induces peripheral DOR analgesic competence through a GRK2-dependent mechanism. Within this framework, paroxetine mimics the inflammatory priming cascade by scaffolding GRK2. Consequently, constitutive phosphorylation of GRK2 at Ser685 is impaired and chronic GRK2 association with plasma membrane DOR reduced, thus freeing DOR from its incompetent state in sensory neurons. This study provides proof-of-concept that the pathways regulating peripheral opioid receptor incompetence can be targeted to enhance opioid-mediated analgesia in the absence of inflammation. Because paroxetine targets the protein that governs peripheral opioid receptor responsiveness, and does so in the absence of inflammation, we propose that paroxetine may be suitable as a repurposed FDA drug that can be used as a co-therapy with peripherally-restrictive doses of opioids to improve analgesic efficacy in noninflammatory pain conditions. The findings within this study support the preclinical recycling of paroxetine for an unrecognized indication and establishes rationale for clinical trial implementation.

## Synthesis

Reviewing Editor: Miriam Goodman, Stanford University Decisions are customarily a result of the Reviewing Editor and the peer reviewers coming together and discussing their recommendations until a consensus is reached. When revisions are invited, a fact-based synthesis statement explaining their decision and outlining what is needed to prepare a revision will be listed below. The following reviewer(s) agreed to reveal their identity: Wendy Walwyn.

The authors describe a series of studies defining a novel role of paroxetine in activating delta opioid receptors (DOR)by inhibiting G-protein Receptor Kinase 2, GRK2. The authors propose that the resulting analgesia could open up a novel role for paroxetine, a selective serotonin reuptake inhibitor typically used to treat a range of mental disorders. The authors show that paroxetine reduces GRK2 association with DORs, as shown by co-immunoprecipitation. Paroxetine also increases DOR inhibition of K+-evoked Ca2+ influx in neuronal cultures. Two control compounds are also studied in these experiments, a GRK2 inhibitor and fluoxetine, a similar SSRI but with no known GRK2 effects. The involvement of GRK2 is further defined by over-expression and inhibition of this molecule and effects on DOR inhibition of K+-evoked Ca2+ influx defined. Finally, the authors assess the ability of a pre-treatment with systemic or peripheral paroxetine to allow DOR agonist induced reversal of PGE2-induced allodynia. This effect does seem to have an inverted U-dose response effect. Finally a series of protein studies show that paroxetine reduces GRK2 phosphorylation, membrane translocation and DOR-GRK2 co-immunoprecipitation. This is reported as a peripheral effect. This work is based on prior work showing that paroxetine inhibits GRK2 and that the relative quiescence of peripheral DOR activity may be a function of constitutive GRK2 association with this receptor.

This is an elegant and conclusive study combining these two known findings as a preclinical proof of concept series of experiments that could support further consideration as a clinical intervention.

Editor’s and reviewers consensus comments

1. Figure legends containing detailed reports of paired posthoc tests (e.g. Figure 3) are very difficult to digest. Please streamline the report.

2. The number of technical and biological replicates are not clearly indicated in all figure legends. In the legend to Figure 1, the number of trials is reported, but not the number of cells studied. In Figure 2, the number of cells is reported but not the number of animals. If all of the cells studied were isolated from a single animal, then additional data are needed to have confidence in the results.

3. Statistical reporting and statistics. Results are analyzed using ANOVAs and the overall p values emerging from the ANOVAs are reported. Please also report the F statistic and degrees of freedom. [Editor’s note: You might also consider a different statistical approach. A fundamental assumption of an ANOVA is that the measurements are normally distributed. However, with small n, it is difficult to be determine normality.

4. CMPD101 is also a GRK2/3 inhibitor, but there are some relative differences in effects of paroxetine and CMPD101 in some of these experiments. Please comment on these differences in the results and discussion.

5. DPDPE is not particularly DOR-specific. Mu opioid receptors (MORs) can also be constitutively silenced by GRK2 and affected by paroxetine. Additional experiments using a MOR agonist or antagonist would provide more information about specificity.

6. It looks like all the experiments in this study are performed cells and tissues obtained from male rats. Please indicate this in the abstract and introduction. Please also discuss limitations of the study that arise as a result of this experimental design choice in the discussion.

7. There are several typos in the text that need attention, here are two examples:

a. P10; Please correct: “in beyond vehicle-treated levels.”

b. P8: (Brackley et al., 2016; Brackley et al., 2017),22-23.

8. Please define “CAP", which is assumed to be capsaicin but is not written out anywhere in the manuscript.
