# Supplementary figures and images for: Paroxetine Increases δ Opioid Responsiveness in Sensory Neurons
Source: eNeuro. 2022 Aug 1;9(4):ENEURO.0063-22.2022. doi: 10.1523/ENEURO.0063-22.2022 (PMC9347309; doi:10.1523/ENEURO.0063-22.2022)

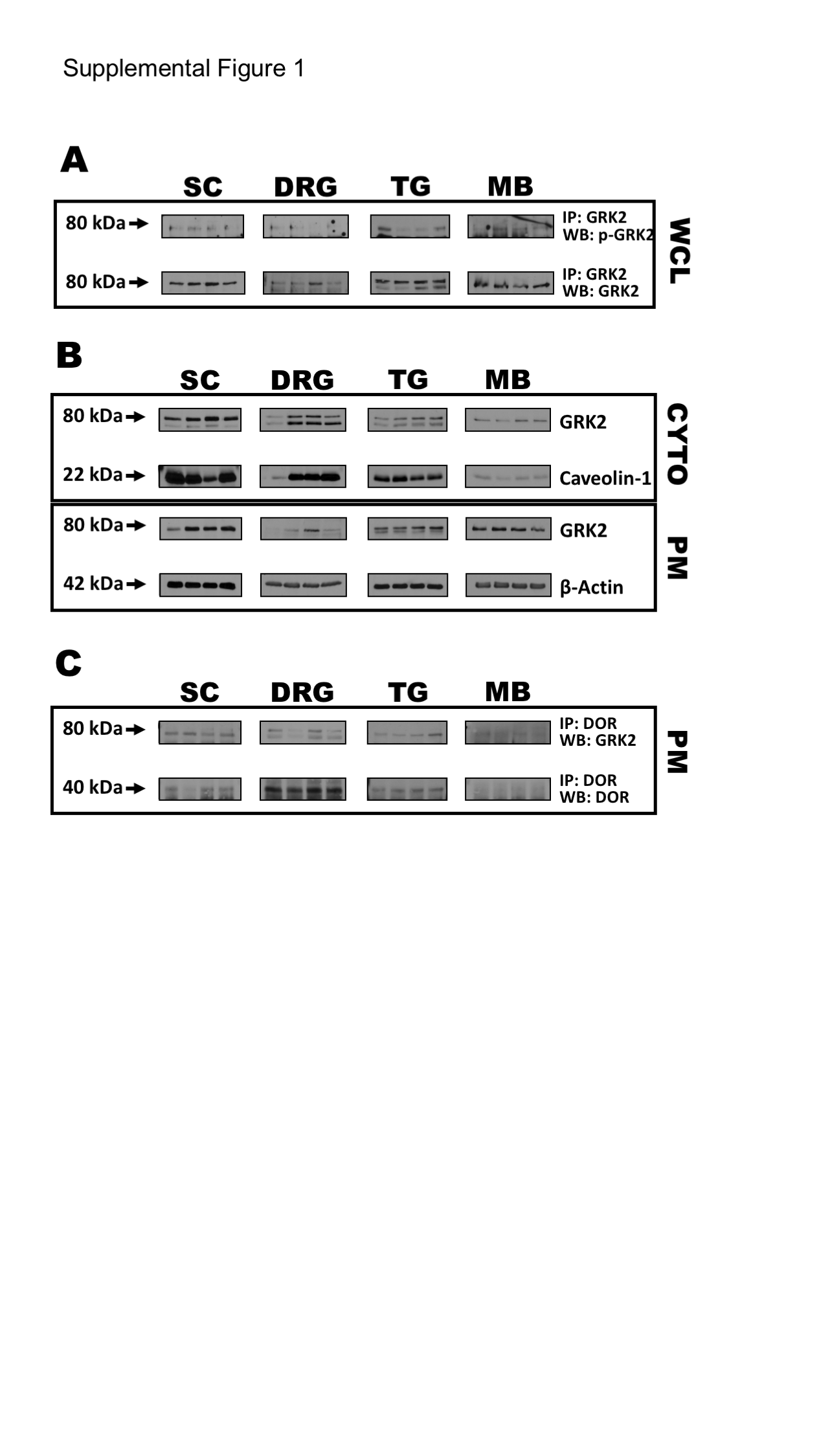

Supplement: Extended Data Figure 3-1 — A, Representative WB images used to calculate values in Figure 3E. B, Representative WB images used to calculate values in Figure 3F. C, Representative WB images used to calculate values in Figure 3G. Download Figure 3-1, TIF file. [file enu-eN-NWR-0063-22-s02.tif]
